# Contribution to understanding the influence of fires on the mercury cycle: Systematic review, dynamic modelling and application to sustainable hypothetical scenarios

**DOI:** 10.1007/s10661-022-10208-3

**Published:** 2022-08-24

**Authors:** Ahinara Francisco López, Eric G. Heckenauer Barrón, Pastora M. Bello Bugallo

**Affiliations:** School of Engineering, Universidade de Santiago de Compostela, Av. Lope Gómez de Marzoa, s/n, 15782 Santiago de Compostela, Spain

**Keywords:** Forest fires, Mercury cycle, System dynamics, Multimedia modelling, Wildfires policies, Environmental indicators

## Abstract

**Abstract:**

Mercury (Hg) mobilization and accumulation in the environment is directly related to forest fires. Biomass burning accounts for about 13% of the total contribution of Hg from natural sources. The aim of this work is to contribute to the knowledge of how wildfires modify mercury compounds behaviour and the effects it has in the Hg cycle, based on a systematic bibliographic review and analysis. Systems dynamics is an adequate focus to analyze the mobilization of Hg due to wildfires, which meets all the requirements to be studied by multimedia modelling. The development and application for the first time of a dynamic multimedia model of Hg taking into account specifically the influences of wildfires is one of the novelties of this work. Different scenarios show that an increase in the number of fires will consequently increase the mercury emitted into the atmosphere, modifying its natural cycle, producing a long-term modification of Hg compositions and concentrations in the different media. Hg movement caused by wildfires can cause complications in living beings and alter the ecosystems. This study found that the Hg soil content could as well be an indicator to measure the impact of fire on the environment. This model can also be generalized to conduct additional studies under comparable conditions, helping to understand the importance of forest fires in global Hg cycles.

**Graphical abstract:**

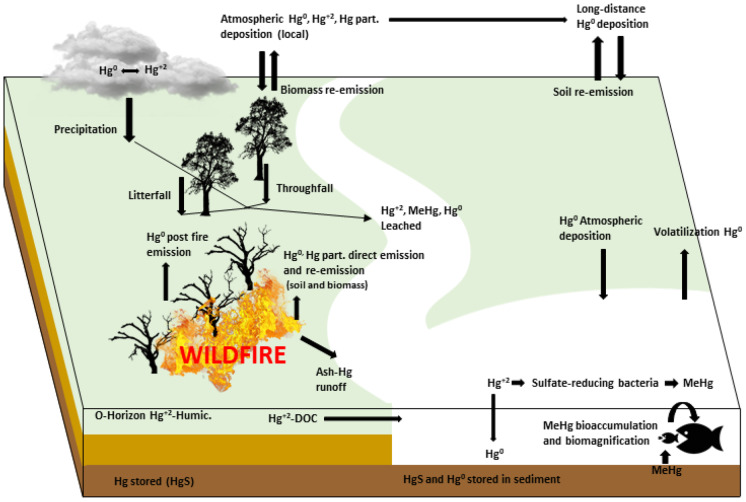

## Introduction

The future evolution of climate, land use and land cover and Hg anthropogenic emissions are all important factors affecting Hg wildfire emissions in the coming decades (Kumar et al., 2018). Wildfires are a significant source of Hg contamination. Impacts from recent climate-related extremes, such as wildfires, reveal significant vulnerability and exposure of some ecosystems and many human systems to current climate variability. For countries at all levels of development, these impacts are consistent with a significant lack of preparedness for current climate variability in some sectors (IPCC, 2014). Ongoing and projected increases in forest wildfire activity due to climate change will increase atmospheric mercury emissions, contributing to the anthropogenic alteration of the global mercury cycle and exacerbating mercury toxicities for food chains. Mercury can pose health risks to humans and animals; human exposure to Hg is predominantly through consumption of fish (Canuel et al., 2009). An increase in methylmercury downstream of recent fires can increase the bioaccumulation which could affect multiple species in a given ecosystem including humans (Sever, 2021).

Climate projections predict larger, more frequent, and more severe wildfires which will result in correspondingly larger Hg releases to the atmosphere and redistribution of Hg over high latitudes (Turetsky et al., 2006). Therefore, it is essential to know how fires modify the mercury cycle. Considering the importance of Hg fluxes to different media and the subsequent danger of MeHg concentrations in fish, there is a need to characterize Hg cycling and transport.

Among natural sources, mercury emissions from biomass burning have recently received growing attention due to the significantly contribution to the regional atmospheric mercury budget (Cinnirella et al., 2008). Wildfires are also important in the global and regional Hg cycle through the mobilization of sequestered Hg reservoirs. Soils are important dynamic pools in the mercury cycle (Homann et al., 2015). By mobilizing mercury from relatively immobile soil and vegetation pools into the atmosphere, wildfires also have a direct impact on the emission and deposition processes of mercury (Engle et al., 2006). Forest ecosystems are an important sink (HgS) for atmospheric Hg, but they are also a supply of Hg due to biomass burning (re-emission), and the following processes in time, like soil deposition, runoff, and input in the food chain. The relationship of the amount of burnt biomass and surface with Hg emissions to the atmosphere suggests that the control of fires may well reduce Hg releases to the atmosphere (Cinnirella et al., 2008).

Mercury is an element that can be found in different chemical forms in all media, such as water, soil, atmosphere, and biota, and it moves from one to another over time; then it meets all the requirements to be study by multimedia modelling. Complex interactions among interacting forces can be described through dynamic models to capture their cause-effect interrelationships; the models may be run and analyzed to portray reality (Ford, 1999), and policy solutions may be assessed for viability (Bala et al., 2017). System dynamics is an adequate focus to analyze this case. Then, wildfires as complex phenomena involving many factors can be better understood by simplifying those through multimedia dynamic modelling. These models are valuable for describing environmental systems, and they can also be generalized to conduct additional studies under comparable conditions. Dynamic models are a critical tool for understanding the sources, processes, and fate of Hg. However, there has been very little effort towards dynamically modelling the mercury mobilization and accumulation in wildfires. The use of tools such as dynamic modelling to study this phenomenon is novel in this study. Until now, dynamic models of mercury have been carried out, but never relating them to forest fires. Several models have been developed to characterize Hg fate and transport in natural and semi-natural environments. Models exist for the different media, but none of them includes all the different natural media. Most of them are not suitable for dynamic modelling. Models allow us to obtain information without the need to have direct access to it, little investment of time and material is needed.

The hydrological cycle in nature is a conceptual model that describes the storage and movement of water between biosphere, atmosphere, lithosphere and hydrosphere (Eslamian et al., 2018). This cycle is closely related to the Hg cycle. Other elements, such as phosphorus, were used to describe the biogeochemical cycle dynamically (Emelko et al., 2016). The study compares its variations in burned and unburned sections; to see how phosphorus concentrations are modified. Ostad-Ali-Askari et al. (2017) carried out an investigation of nitrate pollution in groundwater of marginal area of Zayandeh-rood River, Isfahan, Iran, which was investigated using ANN models. There are other studies which used the modelling tool to study Hg related to forest fires. Cinnirella et al. (2008), estimated mercury emissions from forest fires in the Mediterranean region based on RS data, by using the Moderate Resolution Imaging Spectroradiometer (MODIS) datasets. The global Hg chemistry model, ECHMERIT, is used to investigate the annual variation of Hg emissions, and the geographical distribution and magnitude of the resulting Hg deposition fluxes from biomass burning (not specifically forest fires) by De Simone et al. (2015). An inverse model is used by De Foy et al. (2014), to estimate the sources of mercury at an urban site, including a transport model (CAMx) and emission scaling factors for emissions from forest fires and lake surfaces. Another Hg wildfires emission inventories and models are used in other works to study Hg related to forest fires from different perspectives. INCA-Hg is a dynamic model of Hg cycling that estimates Hg fluxes from the terrestrial to the aquatic environment (Futter et al., 2012).

This work aims to contribute to the knowledge of mercury compounds’ behaviour due to wildfires and the effects on the Hg cycle by conducting a systematic bibliographic review and analysis. The analysis of the system is completed by the development and application for the first time of a dynamic multimedia model of Hg considering specifically the wildfire influences. As part of this objective, validation, sensibility analysis and policy analysis are conducted to build confidence in the dynamic model and to prove its usefulness in this kind of study. Finally, this work aims to applicate the dynamic model to sustainable hypothetical scenarios to improve decision-making in this context.

## Material and methods

For the review, a systematic search of literature in Web of Science Data Base was conducted to study the analysis of mercury compounds behaviour due to wildfires. System dynamics is an adequate focus to analyze the mobilization of Hg due to wildfires in the environment, and it meets all the requirements to be studied by multimedia modelling.

### The analysis of mercury compounds behaviour due to wildfires

This part includes data collection on the subject matter, analysis and understanding of the mercury behaviour in the environment, the cycle, as well as data from wildfires. In order to compile all available information on the subject, systematic research of literature in the Web of Science for the keywords “wildfire” and “mercury” was conducted. Once data was gathered from each publication, the actual cycle of mercury affected by forest fires was explained in a synthesized way. The information obtained about the mercury cycle gave us the numerical data to create a useful table that shows the figures for the dynamic models.

### Modelling

“System dynamics” is a computer-aided approach to policy analysis and design. It applies to dynamic problems arising in complex social, managerial, economic, or ecological systems in any dynamic systems characterized by interdependence, mutual interaction, information feedback, and circular causality (SDS, 2019).

The method uses computer modelling to focus our attention on dynamic behaviour. Computer simulation is effective when it helps us understand the impact of time delays and nonlinearities in the system (Ford, 1999). Different software is available to build system dynamic models. In this case, *Vensim PLE* (Ventana Systems, 2021) software is used to implement the model building a flows and stocks diagram and characterizing these variables with optimized data and equations which are executed by the software. The values used in the dynamic problem are taken from a systematic review. Three stages of a system dynamic model are developed progressively increasing the complexity, where all the reservoirs in which mercury is mobilized are represented. A first order model, second order model and final model are simulated. A causal loop diagram is elaborated from the final model. The evaluation and interpretation of the model includes the model validation, sensitivity analysis and policy analysis. The model validation is made by comparing model outputs to historical Hg concentrations. The sensitivity analysis identifies the key parameters driving model results. Finally, a policy analysis is carried out with the model to provide guidelines for the improvement of the dynamic system from a sustainable point of view. The application of the validated model to various scenarios is used to establish improvements in these systems.

Models are usually built in a step-by-step manner. The summary of steps followed in this paper are a combined method of the proposals of Ford (1999) and Bala et al. (2017):

- Get acquainted with the system and the problem. Identify the problem and formulate the mental model in the form of a verbal description (problem identification/conceptualization) and develop a dynamic hypothesis to account for problematic behaviour in terms of causal loop diagrams and stock and flow structure of the system. The first step to follow is to get acquainted with the system. The scope of the study should be clearly stated to identify the causes of the problem for a clear understanding of it.
- Be specific about the dynamic problem. Create a basic structure of the causal diagram from the verbal model. System dynamics help us think about how a system changes over time. The length of time used, the time horizon, must be long enough to show the dynamic changes.
- Construct the stock and flow diagram and draw the causal loop diagram. Augment causal loop diagrams into system dynamics flow diagrams and translate the system dynamics flow diagrams into *Vensim PLE*. The choice of this software is since it is easy to use, and that it is freely accessible.
- Estimate the parameter values.
- Run the model to get the reference mode. Validate the model, analyze the sensitivity and the policy.
- Test the impact of policies and applications of the model. It is important at the outset of any project to specify the purpose of the model.

## Development and results

### Analysis of mercury compounds behaviour due to wildfires

After the bibliographic search, 24 results about Hg in wildfires were obtained (Table 1). The research shows that few studies characterizing the distribution of Hg in ecosystems or examining mercury in burned areas are available.

**Table 1:** Collection of data from the different stages of the mercury cycle when it is disrupted by a wildfire

**References**	**Localization**	**Hg soil**	**Hg atmosphere**	**Hg water**	**Hg biota**	**Comments**
Amirbahman et al. (2004)	Acadia National Park, Maine, USA 1*	THg unburned soil: 0.18 kg/ha THg burned soil: 0.13 kg/ha [Hg] O Horizon: unburned 134 ± 48 ng/g dry weight, burned 103 ± 23 ng/g [MeHg]: unburned 0.07 ± 0.07 ng/g (0.16 g/ha) burned 0.20 ± 0.13 ng/g (0.30 g/ha) Average litterfall Hg: Unburned 52.9 ng/g, burned 39.7 ng/g	Average 7.9 µg/m^2^ yr			1*: Cadillac Brook watershed burned in 1947 Hg is more mobile in the unburned watershed
Bank et al. (2007)	Acadia National Park, Maine, USA				Proportion of MeHg to Hg in (phytoplankton and zooplankton) 12–21%	
Burke et al. (2010)	Soils collected from Southern California after wildfire and seasonally during a year (Semi-arid system)	Postfire Hg[] average: 18.5 ng/g Prefire Hg []average: 19.0 ng/g 1 year after: 50-70 ng/g higher		[Hg] Leachate: Burned 0.69 ng/g 2.14%Hg leached Unburned:0.20 ng/g 3.89% Hg leached		
Campos et al. (2015)	Ermida and S. Pedro do Sul, Portugal 1*	Loss of 1.0–1.1 g /ha 2* ^burnt soil 70% of Hg contained in the non−^burnt soil	30% of the Hg released by fire 1.1 g /ha released to the atmosphere	0.5 g/ha introduced into aquatic systems		1*: 5361 ha were covered by *Eucalyptus globulus* Labill an *pinus pinaster* Ait Samples: 4–14 weeks after fire 2*: between 150-550ºC
Cinnirella et al. (2008)	Mediterranean region		Total: 4.3 Mg/year Spain 322.9 kg/year			Emission factor 112 ± 17 µg/kg Monthly atmospheric Hg emissions for 2006
De Simone et al. (2017)	Emissions from global inventories 2013	Hg that deposits over land 33%	30% emitted as Hg particulate	^Total Hg that deposits over the sea^ 67%		
Engle et al. (2006)	Sierran forest site California, US	Mineral soil more than 90% of the total ecosystem reservoir Litter: preburn 2.77 ± 1.75 g/ha Postburn 0.16 ± 0.23 g/ha Mineral soil: preburn 42.7 ± 12.9 g/ha Post burn 41.5 ± 21.4 g/ha	Emissions 2.2 to 4.9 g ha^ − 1^ 1*		Tree: preburn 1.63 ± 0.48 g/ha postburn 1.05 ± 0.43 g/ha	1*: litter and vegetation, the most important sources Ecosystem: preburn 47.1 ± 12.3 g/ha postburn 42.7 ± 21.8 g/ha
Finley et al. (2009)	Mount Bachelor Observatory, Oregon 2005–2007		Hg particulate represents 15% of the total Hg released from wildfires. Hg ^0^ > 95% Average PHg peak 14.67 pg/m^3^			
Fraser et al. (2018)	Canada 2010–2015	Average annual mercury deposition 0.3–2.8t (average)	Total biomass burning emissions of Hg 6-14t (average)			
Friedli et al. (2003)	Washintong State, USA 2001		Total gaseus Hg 94.5% Total particulate Hg 5.4% Emission Factor average: (108 ± 57) × 10^**−**6^ g/ Kg Hg released 2001 Washintong State and Oregon: 0.715 ± 0.27 t/yr 1*			1*: Area burned: 605,867 acres/yr
Homann et al. (2015)	Southwestern Oregon, USA	_Soil Hg loss 0,.4–1,.6 mg/m_2				During two decades (samples 1 year and 9 years post fire)
Howard et al. (2019)	North-West of Tasmania, 2016	[THg] unburned soil: 29.4 ± 17.7 µg/kg Burned soil: 49.3 ± 29.0 µg/kg	Hg emission factor 28.7 ± 8.1 µg/kg dry fuel, emission ratio of GEM_**CO**_ 0.58 ± 0.01 × 10^–7^		[THg]unburned: leaves 47.8 ± 3.5 µg/kg, bark 24.5 ± 4.0 µg/kg	
Johnson et al. (2007)	Acadia National Park, Maine, USA. 1*	Throughfall [Hg]: burned 14.2 ng/l unburned 18.8 ng/l Hg deposition flux: burned 30.8 ng/m^2^ day, unburned 41.1 ng/m^2^ day Throughfall[MeHg]: burned 0.07 ng/l, unburned 0.10 ng/l MeHg deposition: burned 0.16 ng/m^2^ day, unburned 0.30 ng/m^2^ day				1*: Samples collected 2000
Kahl et al. (2007)	Acadia National Park, Maine, USA			Total Hg in streamwater (ng/l): 1999, burned 0.59 unburned 1.29; 2000, burned 1.17 unburned 2.14; 2001, burned 0.70 unburned 1.48; 2002, burned 0.44, unburned 4.05; 2003, burned 0.93, unburned 3.36. MeHg (ng/l) burned and unburned 2000- 2001: 0.04		
Kelly et al. (2006)	Moab Lake, Alberta, Canada			MeHg[] 0.10 ng/L THg[] 2.72 ng/L year 2000 []declined 0.03 ng/L MeHg and 0.55 ng/L THg in 2001 THg export to water 29.7 mg/ha MeHg export to water 0.33 mg/ha	Lake trout muscle: 215 ng/g 1994, 250 ng/g 2001, 391 ng/g 2003	Prefire 1990s, postfire 2000 (43 day period in September)
Ku et al. (2018)	North Califonia 2015 1* (Wragg and Rocky Fires)	Hg ashes 4-125 ng/g derived from vegetation White ashes Hg losses of ≥ 90%, Black ashes 34–83%		< 1% is expected to be released to aquatic environments		1*: samples 3–5 weeks after fire without rainfall. Black and white ashes sequester aqueous inorganic Hg but not gaseous elemental Hg
Melendez-Perez et al. (2014)	Brazil in Southwest Amazonia	Litterfall and O-horizon 78% of released Hg. Hg[] before burning 84–92 ng/g. Hg burden before 2.8 g/ha, after 0.2 g/ha. [Hg] in ashes 12 ng/g	Total: 4.1 ± 1.4 g /ha Total soil Hg emission 3.0 ± 1.4 g/ha		14% released from live biomass. Tree leaves 33 ± 18 ng/g, 6 ± 5 ng/g in bark and 1.5 ± 1.5 ng/g in wood	Fuel burning loading, emission factor 40–53 µg/kg
Navratil et al. (2009)	Czech Republic	Total soil Hg pools at the unburned area 126-208 g/ha	Complete combustion (4039t)caused volatilization of 1.34 ± 0.007 kg. 2006 Emissions 75.1 g/ha. 2006 26.7 kg/year 2000–2006			
Nelson et al. (2007)	Acadia National Park, Maine, USA	Watershed accumulation burned soil 9.0 µg/m^2^ yr, 95%THg retained Accumulation unburned soil 8.9 µg/m^2^ yr, 87% THg retained. 1*		Total [Hg] average in throughfall burned watershed 14.2 ng/l, unburned watershed 18.8 ng/l Average [MeHg] in precipitation: burned 0.07 ng/l, unburned 0.10 ng/l. MeHg export(to water) burned soil 0.04 µg/m2 yr, unburned 0.06 µg/m^2^ yr		1*: Do not include potential volatilization losses
Patel et al. (2019)	Acadia National Park	[Hg]: 1999 Unburned 134 ± 20 ng/g, burned 103 ± 9 ng/g. 2015 Unburned 281 ± 11 ng/g, burned 176 ± 26 ng/g				Comparisons between 1999 and 2015
Peckenham et al. (2007)	Acadia National Park, USA 1*			Hg stream water samples: Median of 1.2 ng/l Hg in wetland streams 2.8 ng/l Hg in upland streams 1.0 ng/l 2*		1*: Cadillac Brook watershed burned in 1947 Samples 1999–2000 2*: It cannot be proved that the differences are due to the wildfire
Pirrone et al. (2010)	Global scale		675 Mg/year 1* 13% of the total contribution from natural sources 9% of the global emissions 28% of emissions from land			1*: annual average for the period 1997–2006
Sheehan et al. (2006)	Acadia National Park, Maine, USA. 1*	Litter [Hg] mean 46.9 ± 1.9 ng/g Annual Hg flux in litter: burned soil10.0 µg/m^2^, unburned soil 10.1 µg/m^2^ Wet deposition Hg: burned 9.4 µg/m^2^, unburned 10.2 µg/m^2^		Annual Hg flux to stream water: burned soil 0.4 µg/m^2^, unburned 1.3 µg/m^2^		1*: Samples collected 2003- 2004
Webster et al. (2016)	Western United states for 2000–2013 period		3100 ± 1900 kg/year Emission factors: high severity 58- 640 µg/kg fuel Low severity 18-34 µg/kg fuel		Hg[] leaves 19-28 µg/kg	

Mercury appears in nature in different oxidation states and organic and inorganic compounds. It may appear in the gas phase (Hg^0^), ((CH_3_)_2_Hg), as a liquid (Hg^0^), in the solid phase (HgS) and solution ([CH_3_Hg]^+^ and others). Mercury has a high vapour pressure (0.0017 mm of Hg at 25 ºC). It is found primarily in the vapour phase in the atmosphere, where it can remain for at least one year (Johnson et al., 2007). However, residual Hg can remain until 100 years in the soil (residence time) (O’Connor et al., 2019).

Mercury is present in the Earth´s crust at an average rate of 0.05 mg/kg; it is a chalcophile element, appearing fundamentally like simple sulphide (HgS), cinnabar (Macías Vázquez et al., 2009), being the oceans the largest reserves of it. Mercury follows a cyclical dynamic in a natural way influenced by anthropic activities. The biogeochemical behaviour of mercury is complex, involving atmospheric, geological, biological, hydrological, and chemical processes, among others. Besides, industrial activities such as metallurgy and mining alter its natural cycle. Biomass burning accounts for about 13% of the total contribution of mercury from natural sources (Friedli et al., 2003). Organic horizons have more importance than mineral (inorganic) horizons in the Hg biogeochemical cycle because wildfires have the potential to mobilize Hg from biomass but not from the C-horizon (the deeper mineral soil) (Friedli et al., 2003). Mineral soil is the largest mercury reservoir in most ecosystems, and it is less reactive, but litter and vegetation reservoirs play a significantly more active role in the cycling of Hg (Krabbenhoft et al., 2005).

Mercury in foliage results primarily from the accumulation of atmospheric Hg with soils exerting a minor influence (Hanson et al., 1995). It enters terrestrial ecosystems via precipitation (the little flux), litterfall (the most significant flux of Hg in forest ecosystems), throughfall (collected below the forest canopy), and dry deposition (Navratil et al., 2009). Atmospheric Hg accumulates on the surface of foliage via oxidation of Hg^0^ at the leaf surface (where it also can be volatilized), or adsorption of gaseous (Hg^2+^) or particulate Hg at the air–water interface on the leaf surface (Rea et al., 2002). Particulate Hg deposited to foliage can enhance Me-Hg formation (Witt et al., 2009) and this uptake by vegetation is an important channel to enter the food chain. Undeveloped landscapes with dense wetlands and forests generally yield highly favourable settings for converting inorganic mercury, which is relatively unavailable for biological uptake, to methylmercury (Flanagan Pritz et al., 2014). Volatilization and emission of mercury from vegetation, litter, and soil during a wildfire represents a significant return pathway (re-emission) for previously deposited atmospheric mercury (natural or anthropic) (Howard et al., 2019). Most of the emissions are from litterfall and O-horizon; live biomass represents a lower value (Melendez-Perez et al., 2014).

Mercury released from fires to the atmosphere is predominantly in the elemental stage (Hg^0^), which can be transported long distances, or particulate mercury that is deposited locally (Engle et al., 2006). Total post-fire emissions may come to exceed releases of the metal during the fire itself (Melendez-Perez et al., 2014). Wildfires can release to the atmosphere deposited metals on the soil surface, directly by combustion of vegetation and soil organic matter mineralization and leaching from ash-soil interactions (Campos et al., 2015). Ash from wildfires is a very heterogeneous material, which exhibits higher organic matter and Hg relatively to soils. There is a strong affinity of Hg for carbon in ash, also elevated ash Hg concentrations may be due to the sorption of atmospheric Hg (Engle et al., 2006). Also, rainfall has a significant role in the redistribution of Hg; rainfall and runoff are important inputs of contaminants from diffuse sources to rivers and estuaries. The elevated risk of erosion can result in increased delivery of organic or particulate-bound Hg to surface waters in post-fire systems (Burke et al., 2010). According to Campos et al. (2015), the wash-over implies transference of Hg from ashes to soils, also the atmospheric deposition of Hg due to the rain. Other quantities could be lost to the adjacent terrestrial areas or eventually introduced into freshwater systems.

There are still few reliable data on air/surface exchange in aquatic systems. Mercury flux is strongly correlated with relative humidity (Feistel & Hellmuth, 2021). Meteorological conditions (temperature and relative humidity) strongly affect the gas-particle partitioning of oxidized Hg, which acts as intermediate between elemental and the organic Hg compounds (Nguyen et al., 2021). When mercury enters aquatic ecosystems, it is transformed by microorganisms, such as sulfate-reducing bacteria, and chemical processes to methylmercury. Watershed Hg mobilization potential and its bioavailability are controlled by the lability of soil organic matter (Amirbahman et al., 2004). Hg in natural waters is complexed and transported by organic carbon; the more organic carbon there is, the more Hg is transported from soils and wetlands (Peckenham et al., 2007). Acidified freshwater ecosystems with high temperatures and dissolved organic carbon levels facilitate MeHg bioaccumulation and biomagnification (Bank et al., 2007). Wildfires can modify complex interactions that control MeHg accumulation by organisms (Kelly et al., 2006).

Typify the soil, land use, topography and heterogeneity of physical condition is important to be able to estimate runoff (Krisnayanti et al., 2021). Post-fire runoff events can mobilize pulses of MeHg and THg to streams, but (Patel et al., 2019) explain that Hg methylation does not run parallel to THg trends, and this is important given the biological implications of MeHg in the environment. In addition, Hg inputs to lakes due to the effect of fires can increase Hg bioaccumulation (productivity) and food chain length. It affects the structure of aquatic communities and concentrations of contaminants in biota (Kidd et al., 1995).

Synthesizing information from background on the Hg cycle, the necessary information to carry out the modelling is shown in Table 1. The table includes data from different works, many of which from Acadia National Park, whose figures were used in the development of the three models. We have to point out that some data has been obtained or interpreted from other works since it was not available in the consulted bibliography.

### Modelling

Ford (1999) describes a model as a substitute for a real system, being clearer to work with it. Models can describe an environmental system, if it works it can be used to conduct other researches with similar conditions. In this case, a computer simulation model will be used.

Mercury is a heavy metal and a persistent pollutant, which circulates in various forms between the atmosphere, water, sediments, soil and biota. Due to its volatility, it can be transported in the atmosphere over long distances. Furthermore, the organic complex methylmercury can bioaccumulate and biomagnify in living organisms.

The characteristics detailed above show the complexity of this element in nature, so multimedia models are the ideal tool for this type of analysis, they are also especially useful for compounds that have a certain persistence. Models allow to predict the behaviour of the pollutants, and consequently, their distribution, reactivity and persistence in the environment (Silva Gomez et al., 2005).

The physical environment is described as various connected compartments, through which exchanges of matter and/or energy are established. The resolution of the individual balances is simple, and the complexity is progressively increased (Silva Gomez et al., 2005). The dynamic problem is the mobilization of Hg during a wildfire (re-emission) and the fate of Hg compounds in the different media/compartments, and the study approach is system dynamics.

The reference model is based on historical information, taken from the wildfire of 1947 in Cadillac Brook watershed in Acadia National Park, Maine (data included in Table 1, among others). This area was chosen as case study because it has been studied for many years and a lot of research exists dealing with the changes the area underwent after the fire. The long-range transport of atmospheric contaminants is especially relevant in Acadia NP, including toxic trace substances such as trace metals, persistent organic substances, mercury, and acidic deposition (Kahl et al., 2007). The inputs of acids and Hg are well characterized, based on data from the National Atmospheric Deposition Program (NADP) since 1980, and Mercury Deposition Network (MDN) since 1995 (Kahl et al., 2007).

#### A first-order model

The real system relevant variables and the cause-and-effect connections are represented in the computer model by variables and interconnections (Ford, 1999). The key variables in the system and their interconnections are very simple for the first model, increasing its difficulty in the following. Building the flows-and-stocks diagram represents the next step. Stocks and flows are the basic building blocks of system dynamics (Ford, 1999), being the stocks the key variables in the model and the actions of the flow that change the system.

**Fig. 1:**
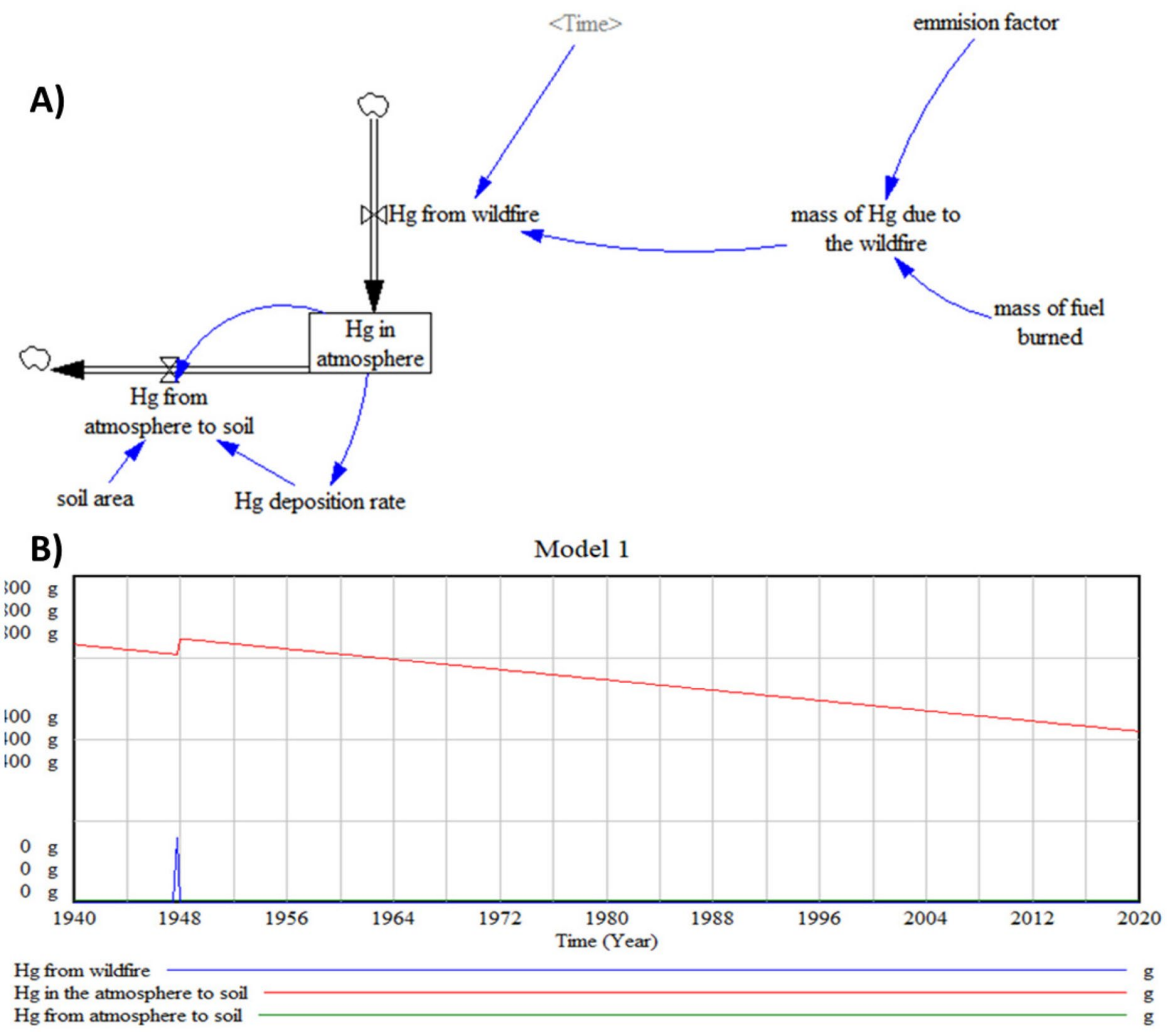
First model: **A** flows and stock diagram and **B** simulation results

Figure 1(A) shows the flows and stocks diagram of the first model to simulate the release of Hg from the wildfire to the atmosphere. Only one stock, Hg in the atmosphere, is considered for the first model. Total mercury is considered without differentiating oxidation states or compounds.

##### Parameter estimation

Cadillac Brook watershed is located on Cadillac Mountain (Maine, USA), within the 31.6 ha burned of the 1947 fires. Webster et al. (2016) described that emission factors for high severity burns ranged from 58 to 640 μg-Hg kg-fuel^−1^. The value used for the air emission factor is 0.0001 g Hg/kg fuel, considering that it was a large fire.

The variable that defines the mass of fuel that was burned in the fire is complex since most of the data found represent hectares burned but not units of weight. For example, fires in temperate forest logging slash can have fuel consumption levels as high as 14 kg/m^2^ (Stocks & Kauffman, 1997). The total biomass loss was 56.87 Mg for the 12.32 ha affected in a wildfire in Mexico (García-Martínez et al., 2017). Some forests in Spain have a vegetal load that reaches 40/50 tons per hectare when above 10 t/ha a fire exceeds the extinction capacity. In the fires of Portugal during 2017, for example, the load was between 23–25 t/h (RTVE, 2018). The biomass obtained if we consider 50 t of fuel equals 1,580,000 kg. According to the references found, this last data is used in the model for the mass of fuel burned in the wildfire.

To determine the mercury that passes from the atmosphere to the soil, the deposition rate of Hg will be used. The deposition rate is 10^–5^ g of Hg in the atmosphere/m^2^·year, for the east of the USA (Mason et al., 1994). To calculate the amount to be deposited in the soil, the deposition rate is multiplied by the area and thus we obtain the deposited Hg. The value used for the deposition flow is obtained from articles, and these values are calculated for each specific case following the calculation method by Cohen et al. (2004).

##### Simulation

Base case simulation covers a period that goes from 1940 to 2020, considering that Acadia National Park (Maine, USA) was affected by a large-scale wildfire in 1947. Initial values for the only existence of this first model in 1940, “Hg in atmosphere”, are calculated considering 2·10^–7^ g/m^3^ (Vinogradova et al., 1985), so the amount of Hg present in the atmosphere in 1940 is 632 g. The length of time used -the time horizon- must be long enough to show the dynamic changes. The fire started in October 1947 and ended in November that same year; therefore, we selected a time step of 0.25 year, which will give a more accurate simulation.

Figure 1(B) shows the results of base case simulation of stock and flows. The amount of Hg in the atmosphere increases immediately after the fire starts and then declines exponentially, as it moves towards and accumulates in other departments.

#### A second-order model

**Fig. 2:**
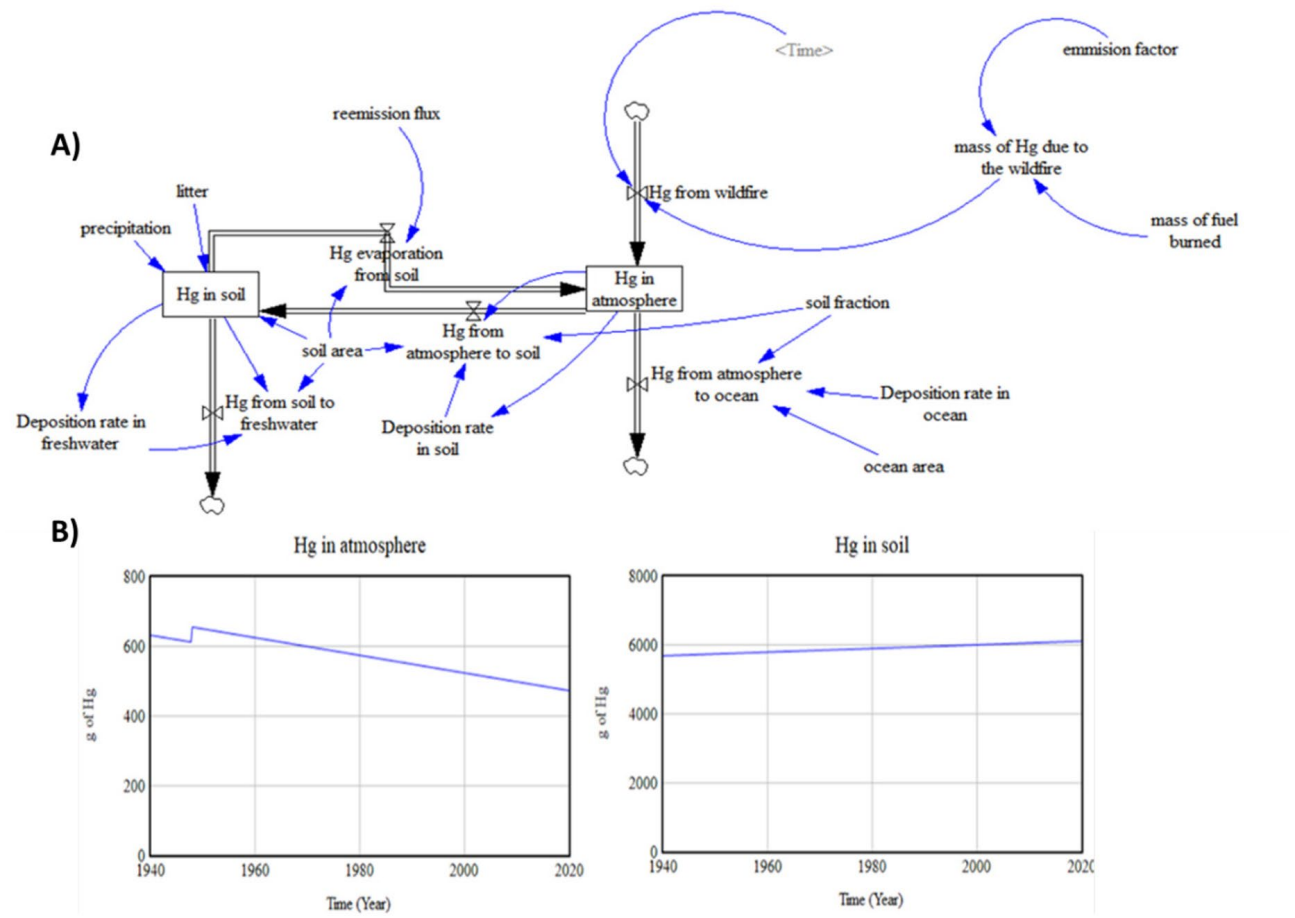
A second-order model. **A** Flows and stock diagram and **B** Hg in the two stocks of the model

Figure 2(A) shows the expansion of the simple model by adding the new stock “Hg in the soil”, a second-order model with two stocks. Mercury deposited in the soil comes from the atmosphere. Besides, a re-emission from the soil into the atmosphere is included. An initial Hg value of an unaltered soil due to wildfires is considered.

##### Parameter estimation

According to De Simone et al. (2017), the total Hg that deposits over the sea is 67% and the Hg that deposits over the land is 33%. So, the soil fraction considered is 0.33. The deposition rate in the ocean estimated is 1·10^–5^ g/m^2^ year (Mason et al., 1994). The ocean area used for this model is 316,000 m^2^.

Hg in the soil is the new existence, soil classification will help set some values. Lyman and Schoodic soils are Spodosols, acidic forest soils characterized by an accumulation of iron (Fe), aluminium (Al), and organic matter in the B horizon. Spodosol (U.S. Soil Taxonomy) is ashy gray, acidic soils with a strongly leached surface layer, which typically form on sandy materials under coniferous or mixed coniferous and deciduous forests. The volume of soil to be considered encompasses the surface of 31.6 ha equivalent to those that were burned and 25 cm deep, because the total Hg concentrations are higher in the O horizon if compared with mineral horizons. It may be primarily attributed to the higher concentration of organic C found in the O horizon. This higher Hg concentration is to be expected because of the strong affinity of Hg towards natural organic matter (Amirbahman et al., 2004).

Sheehan et al. (2006) define in an article that the amount of Hg in the soil is 0.018 g of Hg/m^2^. The soil area considered is going to be like the one burned, 316,000 m^2^. In addition to mercury from the atmosphere entering the soil stock, it also enters as litter and as precipitation. The variable litter has a flux of 1·10^–5^ g/m^2^ year (Sheehan et al., 2006). Precipitation (in this case wet deposition) is defined here as an estimate of annual wet deposition that included throughfall plus wet-only precipitation, the value is 6.8·10^–6^ g/m^2^. Annual Hg export from the soil via stream water to freshwater is 1.3·10^–6^ g/m^2^ (Sheehan et al., 2006). Finally, the re-emission flux will be 2·10^–6^ g/m^2^. Re-emission of Hg following reduction by natural organic matter may, therefore, be an important pathway to be considered in global models, further supporting the need for a process-based assessment of land/atmosphere Hg exchange (Jiskra et al., 2015).

##### Simulation

The simulated response of the new model is shown by Fig. 2(B). The time step established is 0.25. As before, the new model responds, in general, in the same manner as the previous model. But in this case, the Hg in the atmosphere decreases (silts), and it increases in the soil reservoir.

#### The final model

Previous models show how to gradually build up a complete diagram of mercury’s potential pathways through the environment. The final model follows this approach adding Hg concentrations in the ocean and fish. The long half-life of mercury in fish results in a relevant accumulation of Hg in an important source of food which will lead to an increase of contamination risk to living things.

**Fig. 3:**
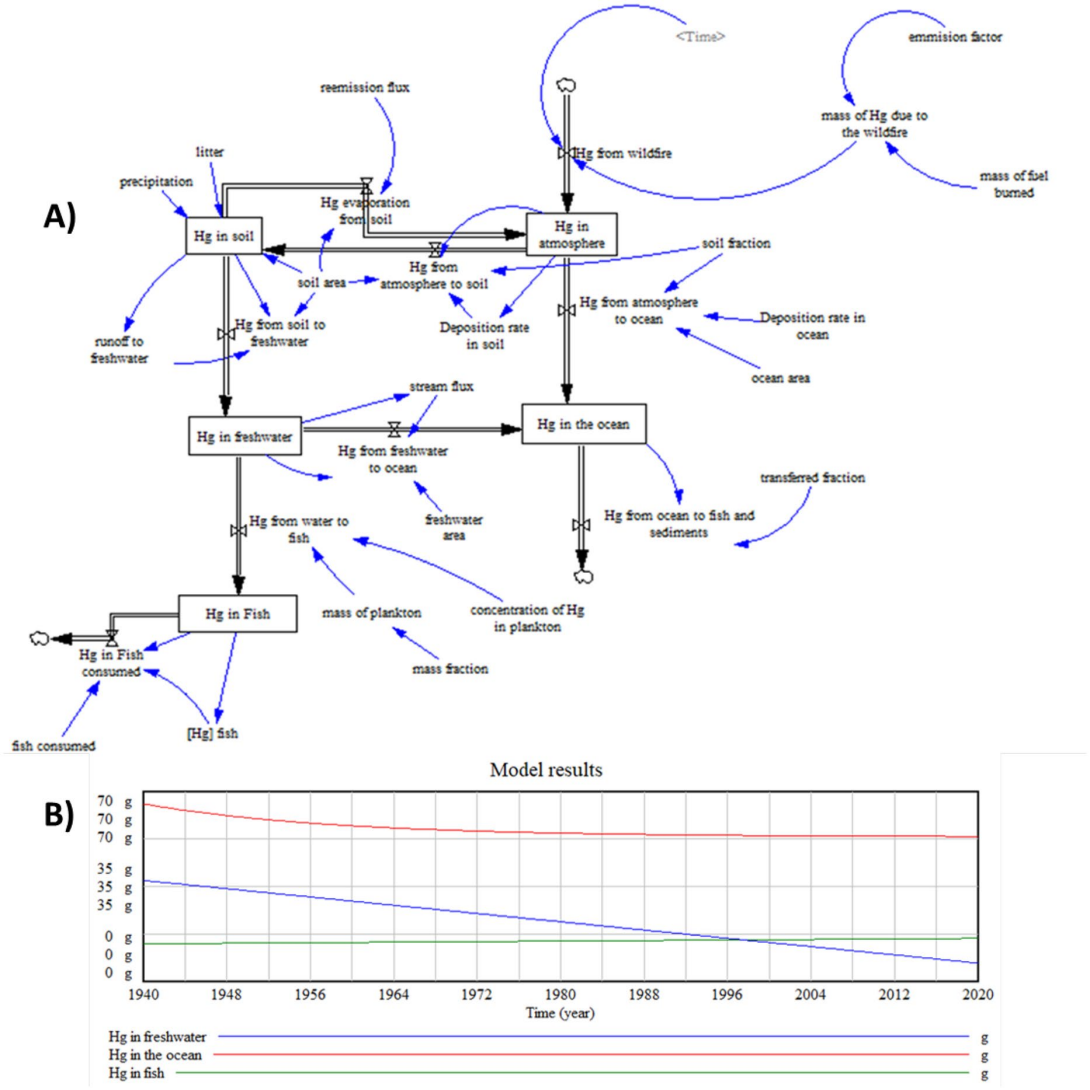
Final model. **A** Flows and stock diagram and **B** simulation results

Figure 3(A) shows the final diagram where five stocks are used to keep track of the Hg in the system. Each stock is measured in grams of Hg.

##### Parameter estimation

Freshwater is present in Acadia National Park in the form of streams, ponds, and wetlands. Lakes and ponds cover about 1052 ha (NPS, 2019). Jordan pond measurements and characteristics are used as a reference for the freshwater stock. It has 76 ha and a water volume of 17,388,000 m^3^. The total amount of Hg in fresh water to be considered for the initial value is 2.14 ng /L (Kahl et al., 2007). To calculate the mercury that passes from freshwater into the ocean, the stream flux is 10^–6^ g/m^2^/year (Sheehan et al., 2006).

For the stock Hg in the ocean, (considering the section of the North Atlantic Ocean), the volume for our stock is 316,000 m^2^ and the deep 150 m. Elemental Hg represents 9–47% of total Hg in subsurface waters (< 150 m). The total Hg concentration in the North Atlantic Ocean is 0.69 pM (1.38·10^–6^ g of Hg) for a depth less than 150 m (Bowman et al., 2020). The initial value is 65.412 g of Hg regarding the superficial part of the ocean for this stock, approximately until the thermocline, the outflow represents the output to depth water and sediments. In relation to trophic structure and deposition, fractions of the total mercury deposited necessary for sustaining levels in fish from coastal regions of the oceans are estimated to be 5.4% (EPA, 1997).

The available data in the bibliography related to mercury in biota, and especially in fish, focus on freshwater fish, taken from the lakes of the park. The concentration of mercury in plankton used is 1·10^–7^ g of Hg/g (Bank et al., 2007). The mean weight of fish in Jordan pond lake is 1311 g (Bank et al., 2007). Taking the fact that the food required for fish maintenance is a percentage of the total weight of fish, thus for an average fish weight of 1000 g, this percentage is 0.8% (FAO, 2020). The average fish density is 1.74 individuals/dam^3^ (Ecohydros, 2011); with an approximate fish weight of 1311 g, the total weight of fish is 39.66 t. To obtain the mass of plankton, we multiplied the total weight of fish by the 0.008 mass fraction. The initial value for the fish stock is 13.88 g of Hg, considering 3.5·10^–7^ g of Hg/g as the mean of Hg for the state (Bank et al., 2007).

The annual fish stocking density yields an average of 0.2 salmon per acre (262.2 g /4046.86 m^2^) in Jordan Pond (The amazing fish-a-metric, 2013). With these values, the grams of fish caught are 49,241.14 g in 760,000 m^2^ and the grams of Hg that come out of the stock, 0.007 g of Hg, considering the 1.4·10^–7^ g of Hg in the fish body (Bank et al., 2007). For all obtained data, salmon has been taken as a reference.

##### Simulation

Figure 3(B) shows the results of the last extension of the model, from 1940 to 2020 with a time step of 0.25. The simulation shows how the Hg present in water eventually accumulates in fish.

##### Stability and vulnerability of the Hg cycle

**Fig. 4:**
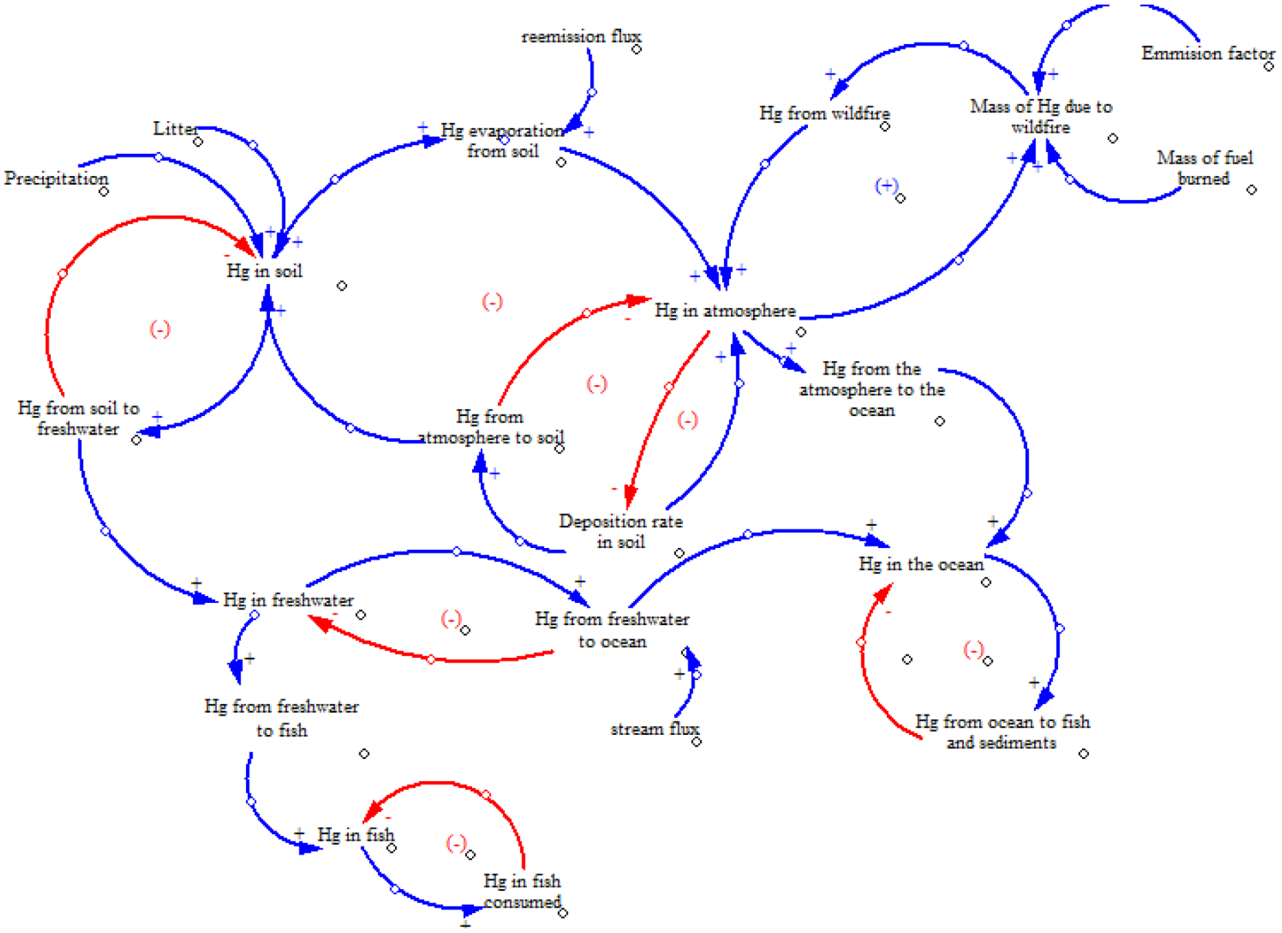
Stability and vulnerability of the Hg cycle (causal loop diagram according to Ford)

Causal loop diagrams are used to describe basic causal mechanisms hypothesized to generate the reference mode of behaviour of the system over time (Bala et al., 2017). Variables in the system are related to causal connections among them. The causal loop diagram of Hg dynamics is shown in Fig. 4, showing the stability and vulnerability of the cycle. Negative feedbacks indicate the stability of the system, on the contrary, positive feedbacks means that instability occurs in the system. Only one positive feedback is present in the causal diagram, when the concentration of Hg increases in the atmosphere, it also increases when it is deposited on the ground, and again it will be emitted with fires.

#### Evaluation and interpretation

The tests for building confidence in the system dynamics model consist of validation, sensitivity analysis, and policy analysis of system dynamics models. Models should be judged, not on an absolute scale but on a relative scale. If they succeed in clarifying our knowledge and insights into the systems for better understanding and management, acceptance of the model is required. (Bala et al., 2017). A model is beneficial if it generates insight into the structure of the real system, makes a correct prediction, and stimulates meaningful questions for future research.

##### Model validation

**Fig. 5:**
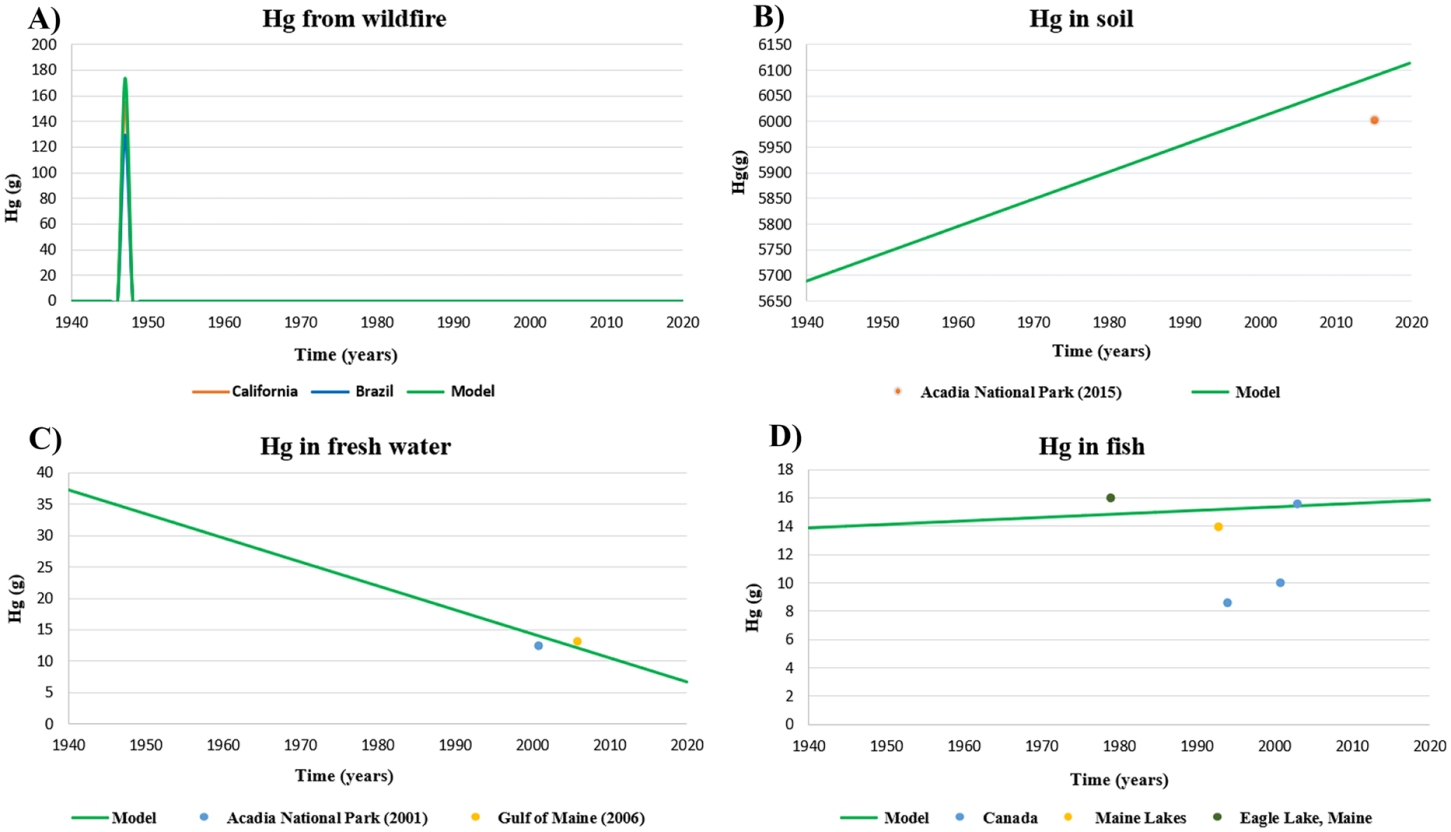
Model validation through historical values

One of the most common and significant tests is to set the inputs to the model at their historical values and see if the outputs match history. In the model, the mercury emitted by the fire is obtained from the fuel mass and the emission factor. Figure 5(A) shows in blue the Hg that is emitted by Acadia National Park wildfire, 173.8 g of Hg or 5.5 g/ha. Red shows the Hg emitted by a fire in California, 4.9 g/ha (Engle et al., 2006), and green the 4.1 g/ha gives off in the Amazon (Melendez-Perez et al., 2014). We can see that despite the differences in the wildfire conditions, the value obtained in the model is common.

Figure 5(C) in our model shows results of 6086 g of Hg in the year 2015. Patel et al. (2019) indicates that the value of mercury measured in 2015 in Acadia National Park in the same place we had set is 190 g/ha, or what is the same 6004 g of Hg for the soil area considered. Furthermore, the same article shows that mercury increases over time in unburned soil. In 1999, there was a Hg concentration of 134 ng/g and in 2015 of 281 ng/g (Patel et al., 2019). Although the value of Hg obtained in 2015 is not the same, it is very close to the data in the article. As shown in Fig. 5(C), the trend in both cases is upward.

Another data used in the model to be contrasted with bibliographic data is the deposition rate, which is an input that we introduce. The Hg that leaves the atmosphere is calculated with the deposition rate 1·10^–5^ g/m^2^/year in the model (Mason et al., 1994). Sunderland et al. (2012), considers 1.15·10^–5^ g /m^2^ /year the deposition rate for the Gulf of Maine, where our case study is located. The amount of mercury in freshwater shown in Fig. 5(B) and offered by the model over time coincides at some point with historical values, that is, in 2001, the model has a value of 13.97 g and 12.17 g according to (Kahl et al., 2007). Unlike in the model, in the bibliography, the mercury in Acadia National Park increases over time, this difference may be because, in our model, part of that mercury passes to the fish and the ocean. Sunderland et al. (2012), offer another figure indicating that in 2006 the value of mercury went down to 13.04 g of Hg in the freshwater of the Gulf of Maine.

Mercury in fish increases over time in the model, and we see this rising trend in other papers such as that of Kelly et al. (2006). The Hg in lake trout muscle in Canada in 1994, 2001, and 2003 is pictured in this article, where, mercury levels are rising (8.53 g, 9.9 g, and 15.50 g respectively), last value coincides with the model.

Figure 5(D) also shows the result of Hg in fish, in Maine lakes, with 13.88 g of Hg in 1993 (Stafford & Haines, 1997). This result corresponds to the one in the model, for the same study area. Another example of Maine lakes indicates that there is 15.86 g of fish in 1979 (Akielaszek & Haines, 1981).

The articles and historical data obtained from research in Acadia National Park do not show Hg values for fish, so they cannot be compared with those of the model.

##### Sensitivity analysis

The purpose is to learn if the model´s general pattern of behaviour is influenced by changes in the uncertain parameters (Ford, 1999). To carry out the sensitivity test, it is necessary to select one of the uncertain parameters, change its estimate and simulate again to observe the variations. The feature SyntheSim allows to see the results of simulations superimposed on the model diagrams and instantly updates these displays as you change model Constants and Lookups. Sensitivity analysis provides an opportunity to determine the level of accuracy needed in the estimation of the parameter to make the model valid and useful.

The first parameter tested is the mass of fuel burned, to see whether it causes changes in the value of the Hg in the atmosphere. This parameter was set at 1,580,000 kg in previous simulations. Consequently, three experiments with this parameter ranging from 1,200,000 kg to 1,800,000 kg were simulated. The test results yield the same finding as in the previous test. The model shows the same general tendency. No changes were found in the other stocks.

**Fig. 6:**
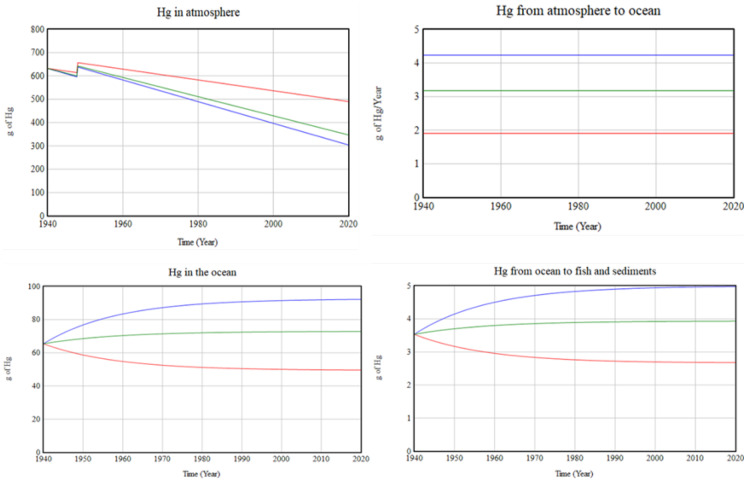
Sensitivity of the Hg in different stocks to changes in the deposition rate. (Run 1 model (green), 1e^−05^ g/m^2^/year; run 2 (red), 9e^−06^ g/m^2^/year; run 3 (blue), 2e^−05^ g/m^2^/year)

However, Fig. 6 shows how variations in deposition flow over time affect the values of stocks that are dependent on this parameter. Considerable change in the simulated behaviour is shown. The same would happen with the rest of the flows or rates that are in the model.

When the deposition value is increased to 2·10^–5^ g/m^2^/year, mercury accumulate in the ocean and the sediments to a greater extent. If a lower value is used, for example, 9·10^–6^ g/m^2^/year, mercury remains longer in the atmosphere and decreases in the ocean.

##### Policy analysis

The purpose of most system dynamics studies is policy analysis, which uses the model to simulate how changes in policies might improve the behaviour of the system. Scenarios based on simulated results can provide guidelines for policy planning and management of complex and dynamic systems.

The first scenario we envision is not very optimistic, because fires are growing more and more virulent and frequent due to climate change. Future changes in land use and land cover may imply changes in vegetation type and density, which at the same time may have a direct impact on future wildfire activity. Drought not only exacerbates wildfire severity and the number of various fuels burned per unit area, but it also dramatically increases fire spread and burn area. Fire frequencies under the 2050 conditions are projected to increase by approximately 27% globally in relation to the 2000 levels (Huang et al., 2015).

2000–2050 climate change could increase Hg emissions by 14% globally (Kumar et al., 2018). Potential enrichment of terrestrial ecosystems in 2050 in response to changes in Hg anthropogenic emissions could increase Hg wildfire emissions globally + 28% and regionally + 19% in North America (Kumar et al., 2018). It has long been recognized that human activity has disrupted the global Hg cycle through fossil fuel combustion, but climate change impacts also may amplify Hg emissions and deposition.

In order to get an approximation of what the future scenario would be in terms of Hg emitted into the atmosphere, we have considered in our model the wildfires that occurred in Maine, USA, in the year 2000, 383 forest fires that devastated 159,041 ha (Maine Forest Service, 2020). The number of fires and hectares burned changes annually without following a clear trend. However, in this case, we assume 27% as the increase in the number of fires compared in 2050 in relation to the 383 wildfires in the year 2000. The model data considers a specific fire in 1947. Now, the same biomass burned (50 t) but this time for 159,041 ha, obtaining 7,952,050 kg of fuel burned in the year 2000. In 2050, it considers 27% more fires. Thus, the burned hectares are 201.98, and the burned fuel 10,099,000 kg. The increase in fires, at the same time, causes an increase in the Hg emitted into the atmosphere.

The model is also useful to make future predictions about changes in forest fire dynamics as it shows the changes that will be obtained in the future according to current policies. A new proposed scenario is focused on soil management. Currently, the impact on soil Hg pools from other widespread forest disturbances such as blowdown and management practices, for example salvage logging, is unexplored. If a wildfire occurs, and inappropriate forest management is carried out, the amount of Hg in the soil can increase and consequently the Hg impact and the movement through the ecosystems will be higher. For instance, according to Mitchell et al. (2012), blowdown increases the mean Hg pool in the forest floor by 223%. If the average of initial Hg in soil is increased in the model to 223% as indicated in the article, the result is a significant increase in the pool of Hg in the soil.

With this practice, the increase of Hg in the soil fixes the Hg, but the problem occurs if there are disturbances that release it. For example, the effect of blowdown-salvage logging-fire led to significant losses in the forest floor Hg pool. Severe fire conditions observed following this succession of disturbances led to much greater consumption of forest floor matter, which likely released more Hg to the atmosphere (Mitchell et al., 2012). Techniques carried out before or after a fire should also be considered in relation to mercury and its possible mobility.

Although the policies applied for the consumption of mercury are not directly related to wildfires, we know that fires can increase the Hg that bioaccumulates in fish and therefore in humans. This scenario generates great concern about the human consumption of contaminated animals, which incorporates the accumulated Hg into the consumer’s tissues, causing nerve and brain damage, and even death. Current health advisories in the U.S. discourage fish consumption for pregnant women and children and recommend limits for adults, based on Hg concentrations in the fish (U.S. EPA, 1997). Mercury contamination is evident everywhere. More than 16 million lake acres and 1 million river miles are under fish consumption advisories due to mercury in the USA, and 81% of all fish consumption advisories issued by the US Environmental Protection Agency are due to mercury contamination (U.S. EPA, 2013).

Fish, bird, and amphibian species exhibit tissue Hg concentrations that exceed acceptable fish consumption advisory limits or risk benchmarks for wildlife, even within national parks such as Acadia National Park (Patel et al., 2019). In 1969, the Food and Drug Administration established 0.5 µg/g as the maximum safety limit of total mercury present in the fish (Elika, 2005). This stricter value was exceeded in the (Bank et al. 2007) article by several fish samples. In 1996, the FDA increased this limit to 1 µg/g due to the results of the national study of the Marine Fisheries Service, showing that the level of 1 µg/g was safe for consumer protection (Elika, 2005). If the limit concentration of 1 µg/1 g and the 49,241.1 g of fish consumed is considered in our model, the grams of Hg that the fish contains in the model (0.007 g) are below the limit value of 0.05 g that meets the regulation.

## Discussion

Studying mercury is a need especially due to its volatilization and toxicity, also due to the potentially harmful impact on human health and the environment. The usefulness of Hg as a tool has already been demonstrated by authors such as Martinez-Cortizas et al. (1999) using it to quantify the effects of human activity. In this study, the results obtained seem to indicate that the amount of Hg in soils is a clear indicator of previous fires and their intensity. Modelling shows that in higher intensity fires, more Hg evaporates, depending also on the type of vegetation present in the area. After a fire, the amount of Hg in the fired soil is much lower than in nearby areas not affected directly by fire but by the mobilized Hg. However, there are certain areas in which anomalies have been found in the soil. For instance, there are areas with higher values than the reference range which can be justified by the presence of Hg in bedrock and specific climate conditions. In contrast, we find others in which the Hg value are lower than expected and which might be the result of volatilization caused by recurrent fires. On this basis, the Hg soil content could as well be an indicator to measure the impact of fire on the environment.

Using the model to check how changes in policies or future predictions will alter the simulation results is its most interesting and relevant contribution. As a matter of fact, if we run the model on the assumption that by 2050 the number of fires will have increased 27%, the result shows that the Hg in the atmosphere will also increase considerably. Consequently, climate change and the increment of wildfires in boreal forests and peatlands which accumulate large amounts of Hg could displace mercury and modify its cycle.

There is some debate about appropriate forest management because, in the case of blowdown and logging, Hg is maintained in the soil, but the amount of Hg released is greater if a wildfire occurs. It may be required to reconsider some practices in which the potential for forest fires to release metals and other substances from stock has not been taken into account. The most relevant policy to consider is the mercury limit in fish, which affects us directly.

## Conclusions and future projections

This work reviewed and analyzed the mercury behaviour modified by wildfires and its effect on the Hg cycle. The global distribution of mercury concludes that the largest deposit is oceanic sediments, followed by the ocean, soil and freshwater sediments, the biosphere (mainly terrestrial biota), the atmosphere and finally fresh water. The analysis has shown for the first time the usefulness of dynamic multimedia models to study Hg mobility in forests affected by fires. The validation, sensitivity analysis and policy analysis to build confidence in the model demonstrated that the model has been constructed from a systems dynamics perspective. The model is useful because it generates insight into the structure of the real system clarifying our knowledge, makes a correct prediction and stimulates meaningful questions for future research.

The projection of four hypothetical scenarios demonstrated that an increase in the number of fires will consequently increase the mercury emitted into the atmosphere, modifying its natural cycle. The necessity to rethink good forest management practices, assessing the condition of forest fires to release metals and other compounds from stocks. The projection showed that peatlands in high areas will become high-risk areas for Hg emissions with the increase in fires and the concern about Hg in fish consumption which can cause complications in living beings and alter the ecosystems. Finally, the study demonstrated that mercury could be considered as an indicator for assessing the extent of fires, as its behaviour and fate are conditioned by the characteristics of fire. This novel contribution can be a very useful tool for future research.

Some of the issues recognized through this work include the need for future research on the Hg cycle considering multiple spatial and temporal scales. The lack of mercury data in forest fires makes the execution of dynamic models difficult. Modelling and analysis of data related to this topic will encourage research to fully understand Hg in forests and wildfires. It will help improve the understanding of the importance of forest fires in global Hg cycles. Due to climate change, a new generation of fires “megafires” are increasing the duration of fire seasons and the risk of more extensive and intense fires, the effects that this new type of fires will cause in the Hg cycle should be investigated further.

## Data Availability

The datasets generated during and/or analyzed during the current study are available from the corresponding author on reasonable request.
